# Mortality Risk amongst Nursing Home Residents Evacuated after the Fukushima Nuclear Accident: A Retrospective Cohort Study

**DOI:** 10.1371/journal.pone.0060192

**Published:** 2013-03-26

**Authors:** Shuhei Nomura, Stuart Gilmour, Masaharu Tsubokura, Daisuke Yoneoka, Amina Sugimoto, Tomoyoshi Oikawa, Masahiro Kami, Kenji Shibuya

**Affiliations:** 1 Department of Global Health Policy, Graduate School of Medicine, University of Tokyo, Bunkyo-ku, Tokyo, Japan; 2 Division of Social Communication System for Advanced Clinical Research, The Institute of Medical Science, University of Tokyo, Minato-ku, Tokyo, Japan; 3 Department of Internal Medicine, Minamisoma City General Hospital, Haramachi-ku, Minamisoma, Fukushima, Japan; National Taiwan University, Taiwan

## Abstract

**Background:**

Safety of evacuation is of paramount importance in disaster planning for elderly people; however, little effort has been made to investigate evacuation-related mortality risks. After the Fukushima Daiichi Nuclear Plant accident we conducted a retrospective cohort survival survey of elderly evacuees.

**Methods:**

A total of 715 residents admitted to five nursing homes in Minamisoma city, Fukushima Prefecture in the five years before 11th March 2011 joined this retrospective cohort study. Demographic and clinical characteristics were drawn from facility medical records. Evacuation histories were tracked until the end of 2011. The evacuation's impact on mortality was assessed using mortality incidence density and hazard ratios in Cox proportional hazards regression.

**Results:**

Overall relative mortality risk before and after the earthquake was 2.68 (95% CI: 2.04–3.49). There was a substantial variation in mortality risks across the facilities ranging from 0.77 (95% CI: 0.34–1.76) to 2.88 (95% CI: 1.74–4.76). No meaningful influence of evacuation distance on mortality was observed although the first evacuation from the original facility caused significantly higher mortality than subsequent evacuations, with a hazard ratio of 1.94 (95% CI: 1.07–3.49).

**Conclusion:**

High mortality, due to initial evacuation, suggests that evacuation of the elderly was not the best life-saving strategy for the Fukushima nuclear disaster. Careful consideration of the relative risks of radiation exposure and the risks and benefits of evacuation is essential. Facility-specific disaster response strategies, including in-site relief and care, may have a strong influence on survival. Where evacuation is necessary, careful planning and coordination with other nursing homes, evacuation sites and government disaster agencies is essential to reduce the risk of mortality.

## Introduction

Following the Great East Japan Earthquake and subsequent tsunami on 11^th^ March 2011[1], [2], a level seven nuclear accident at Fukushima Dai-ichi nuclear power plant caused extensive social disruption and fear in Fukushima prefecture. On the 12^th^ March, shortly after this accident, the Japanese government issued a mandatory evacuation order for those living within a 20 km radius of the nuclear plant and indoor shelter and voluntary evacuation instructions for residents of the 20 to 30 km zone, both of which caused dysfunction in hospitals, clinics, and welfare facilities, and loss of medical supplies[3].

Although all eight nursing home facilities in Minamisoma were located outside the compulsory 20 km evacuation zone, they were all within the 20 to 30 km indoor shelter and voluntary evacuation zone, and all elderly residents of all the homes were voluntarily evacuated irrespective of their individual state of health or care needs, because of the increasing fear of radiation and/or disruption of food, gasoline and medical supplies to this area. Questions about the safety of evacuation of elderly residents are of paramount importance to residents[4]–[7], medical and welfare institutions[4], [8] and, of course, the Japanese government, whose emergency response to the radiation accident is controversial[9]; however, although initial reports suggested a chaotic evacuation with high overall mortality risk[10], there is no detailed understanding of mortality risks associated with the evacuation process[6].

Research on hurricane preparedness in the USA has suggested that evacuation can be associated with an approximately two-fold mortality risk[11], [12]. US nursing homes are required to maintain evacuation plans, though compliance with these plans is not always robust and facility-specific factors may have a strong influence on the mortality and morbidity associated with evacuation[13]; furthermore, experience in the immediate aftermath of Katrina showed that even the best plans of specific facilities may be insufficient to prevent significant morbidity and mortality under a generalized infrastructure collapse, and that support from government both before and after a disaster is essential[14].

Given the importance of disaster- and facility-specific factors in determining the success of disaster plans and the possibility of significant increases in mortality due to evacuation, it is important to conduct detailed epidemiological assessments of the efficacy and safety of evacuation procedures in the aftermath of Japan's triple disaster. Almost a year since the nuclear accident, at the request of the local hospital, we conducted a retrospective cohort survival survey of nursing care home residents in Minamisoma city. Minamisoma city was the only town in Japan that was seriously affected by all three elements of the triple disaster, experiencing infrastructure destruction, significant radiation exposure, and a series of evacuation orders simultaneously. The experience of care home operators in Minamisoma thus paralleled many of the challenges faced in the aftermath of Hurricane Katrina. This is the first detailed assessment of mortality risk associated with evacuation of elderly residents after the Fukushima Dai-Ichi nuclear accident, and offers the first opportunity to explore evacuation-related mortality in detail, as well as a chance to generalize from the specific experience of care home operators in Minamisoma to some of the complex policy issues associated with multiple-cause disasters.

## Materials and Methods

### Ethics Statement

Ethical approval for the study was granted by the ethics committee of the Institute of Medical Science, the University of Tokyo, authorization number 23-61-3038. For monitoring residents' survival, an information sheet on the research objectives and confidentiality of study participation were sent to the care homes' presidents and verbal consent was obtained. The ethics committees agreed that written consent was not required for each care home resident.

### Design, settings, and participants

Five of the eight care homes in Minamisoma participated in this study, representing 62% of all individuals resident in a care home at the time of the earthquake; of those that did not participate, one was unable to due to the loss of all records during the tsunami, and one could not provide sufficient quality evacuation or mortality records. Four facilities are intensive care homes for the elderly, which admit those who have difficulty rehabilitating at home. One facility is a rehabilitation facility for the elderly, which make efforts to enable residents to rehabilitate at home. All elderly residents who had been admitted to the five facilities between 11^th^ March 2006 and 11^th^ March 2011 were included in this study. Information on demographic and clinical characteristics and entry records was obtained from medical records at the facilities, which were recorded by the care practitioners for all residents at entry to the facility. Evacuation history was also recorded by the facilities at the end of 2011 or the beginning of 2012, and this data was collected along with date of withdrawal.

Demographic and clinical characteristics included age at withdrawal or death, sex, and care level, based on the Japanese Category of Condition of Need for Long-Term Care, a number between one and five measuring severity of care needs.[15] This grade is an indicator of severity of disability and does not necessarily indicate health condition[16]. Patients with care level 1 to 4 were defined as requiring low or moderate care and those with care level 5 as requiring high care. Evacuation history consisted of date and site of evacuation recorded separately for each evacuation. Many residents had multiple evacuations, so evacuation distances, indicating the distance between each resident's current location and their next evacuation site, were calculated for each evacuation site as the shortest distance between sites on a public road. Finally, we interviewed facility presidents to obtain further care home-specific evacuation details.

### Data Analysis

To assess the impact of the earthquake on mortality, death incidence density before and after the earthquake was calculated as the number of deaths divided by sum of person-years at risk, which were measured from the date of admission until the end of the study period, death or withdrawal. Person-years of risk were divided into pre-and post-earthquake periods to compare relative mortality and crude relative mortality risk calculated as the ratio of post- and pre-earthquake mortality incidence densities. A seasonal or cohort effect in the data was investigated through visual inspection of a quarterly time-series trend of incidence density. Because Facility 4 lacked data for those who left the nursing home before the earthquake, the total person-years and incidence density before the earthquake was estimated based on the average proportion of person-years for the residents who left the nursing homes before the earthquake in other facilities. Thus the relative incidence density before the earthquake for this facility was an estimate.

Survival probability was assessed using the Kaplan-Meier product limit method, comparison of which was on the basis of the Wilcoxon test and Log-rank test, and plotted with survival curves. Effects of the earthquake itself and mortality risk associated with the evacuation procedures implemented by each nursing home were examined using cox proportional hazards multiple regression. Comparison of survival before and after the earthquake was initially conducted without evacuation history data, to measure the effects of the earthquake on mortality. Evacuation history was explored using only post-earthquake data to estimate risks associated with different evacuation patterns. In both analyses, variables were selected using backward-stepwise model-building. Both analyses included a fixed effect to model unobserved, facility-specific confounders. In the analysis comparing pre-and post-earthquake mortality, a facility-earthquake interaction term was included to test for the possibility of facility-specific moderators of evacuation- or earthquake-related mortality. All analyses were conducted using Stata/MP 11.

The report is presented in accordance with STROBE guidelines.

## Results

### Basic characteristics of care home residents

From 11^th^ March 2006 to 11^th^ March 2011 records were collected for all 596 elderly residents from four of five facilities. Data on residents who had left the facility before the earthquake were missing in one facility. Characteristics of the 715 residents included in this study are shown in Table 1, and interview results with facility presidents and information on facility-specific care level are summarized in Table 2. Other facility-specific evacuation details are described in Table 3. Average number of evacuations indicates the average number of times each facility's residents evacuated.

**Table 1:** Subject characteristics.

Characteristic	Total residents	Number of residents on March 11, 2011	Percentage of total residents
Sex			
Male	192	80	42
Female	523	248	47
Facility Number			
1	144	72	50
2	94	50	53
3	99	50	51
4	119†	69	58
5	259	87	34
Age at death or withdrawal			
50**–**69	30	21	70
70**–**79	110	52	47
80**–**89	339	153	45
90+	236	102	43
Care Level			
Low/moderate	399	224	56
High	316	104	33
Number of deaths by Facility			
1	78	23	29
2	43	12	28
3	52	9	17
4	75	25	33
5	57	6	11

† Pre-disaster data included only those who died.

**Table 2:** Interview results.

Facility	1	2	3	4	5
Type	Intensive care	Intensive care	Intensive care	Intensive care	Rehabilitation
Basic characteristics					
In-house nutritionists	No	No	Yes	Yes	Yes
Medical service	No	No	No	No	Yes
Presence of adjacent hospital	Yes	No	No	Yes	No
Before the initial evacuation					
Short evacuation from tsunami	No	Yes	No	No	No
Continuity of food preparation	Poor	Poor	Good	Good	Good
			(until 17/3/2011)		
Heating	No	No	Yes	Yes	Yes
Time to initial evacuation	19/3/2011	19/3/2011	19/3/2011	15–22/3/2011	17–22/3/2011
During the evacuation					
Suitability of vehicles for evacuation	Poor	Poor	Poor	Good	Good
Support of government	No	No	No	Yes	Yes
After initial evacuation					
Continuity of care	Poor	Poor	Poor	Good	Good
Care quality of evacuation site	Poor	Poor	Poor	Fair	Fair

**Table 3:** Evacuation history by facility.

	Facility Number				
	1	2	3	4	5
Study end	1/12/2011	1/12/2011	1/12/2011	2/2/2012	31/8/2011
Average number of evacuations	2.9	2.6	3.1	1.7	1.0
Average evacuation distance (km) by stage					
Initial	306	303	325	203	242
Second	238	193	261	238	N/A
Third	209	143	223	97	N/A
Fourth	52	145	161	48	N/A

### Examination of possible cohort- and season-effects

**Figure 1:**
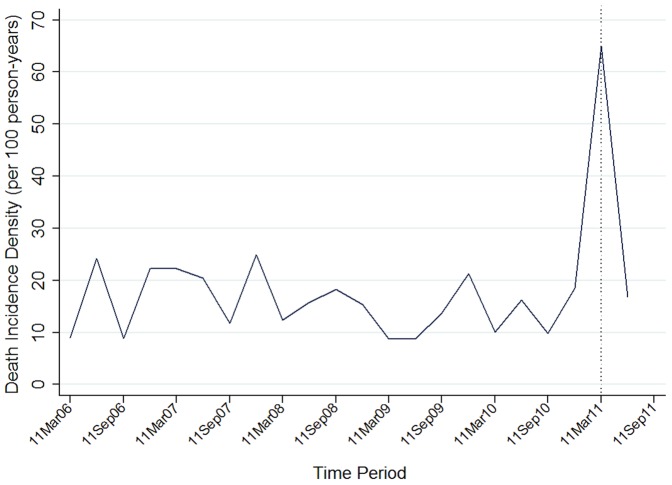
Time series trend of death in elderly homes. Dotted line indicates the time of the earthquake (11/3/2011)

The time-series of quarterly death incidence density for the whole study period is shown in Figure 1. No seasonal effect or upward trend, which might indicate a cohort effect, were observed before the earthquake, suggesting limited observable influence of seasonal or trend effects on the high increase in mortality incidence density after the earthquake.

### Facility-specific mortality risk

Details of the facility-specific relative mortality risk based on the incidence death density are shown in Table 4. A three- to four-fold increase in mortality was observed in three facilities. Facility 5, which did not show a significant increase in mortality, had only one evacuation with a distance of 240 km, suggesting that evacuation number and distance are relevant to the increase in mortality risk; however, Facility 4 had a relatively similar evacuation profile (Table 2), and experienced increased mortality density.

**Table 4:** Facility-specific relative death incidence density.

Facility	Disasters	Population	Death	Incidence Density	Relative Risk	95% Confidence interval
				(/100 person-years)		
1	Before	144	55	14.82	3.78	NA
	After	72	23	56.09		2.22 to 6.26
2	Before	94	31	12.89	3.01	NA
	After	50	12	38.87		1.41 to 6.04
3	Before	99	43	17.36	1.63	NA
	After	50	9	28.24		0.70 to 3.38
4	Before	119†	50	13.95†	3.93††	NA
	After	69	25	54.75		2.36 to 6.57††
5	Before	259	51	15.69	0.98	NA
	After	87	6	15.41		0.34 to 2.29
Combined	Before	596†	230	14.91†	2.68††	NA
	After	328	75	39.82		2.04 to 3.49††

† does not include those who left before the earthquake in Facility 4

†† estimated values

### Probability of survival

**Figure 2:**
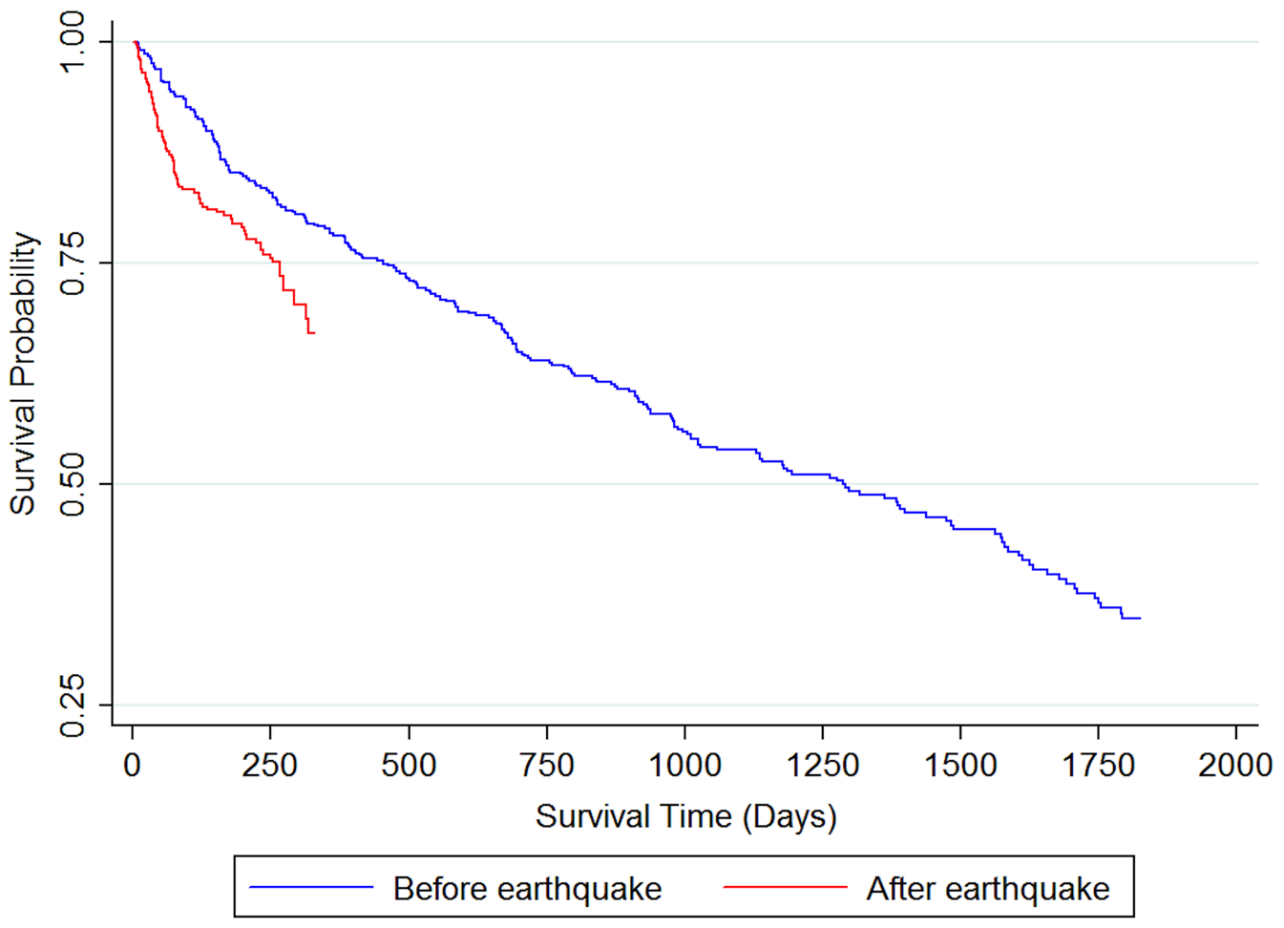
Estimated pre- and post-earthquake survival.

**Figure 3:**
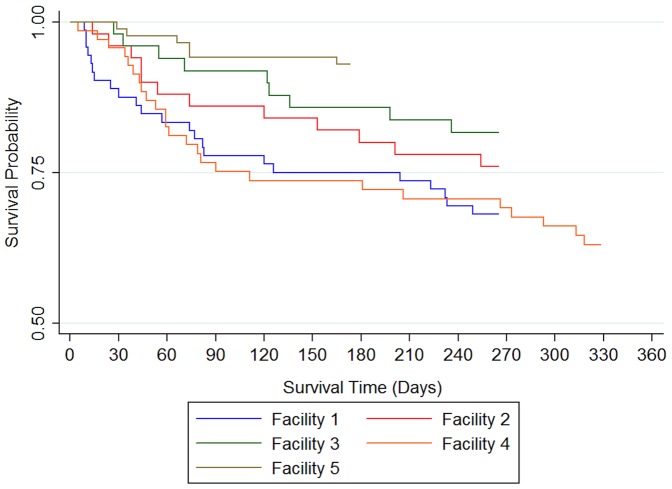
Estimated post-earthquake survival by facility.

Figure 2 shows probability of survival before and after the earthquake for all facilities combined. Analysis time started from the date of nursing home admission and the date of the earthquake respectively. A significant influence of the earthquake on mortality was observed. Facility-specific probability of survival after the earthquake is shown in Figure 3, plotted against analysis time from the date of the earthquake, and shows a large difference between Facility 5 and Facility 1. Facility 5 evacuated once, approximately 240 km distance, while Facility 1 experienced about three evacuations ranging in distance from 200 to 300 km, suggesting that this differential mortality might be explained by the influence of long and repeated evacuations. Facility 4, however, also had high mortality compared with Facility 5 even though their evacuation profiles were relatively similar. This might indicate that other facility-specific evacuation processes are associated with these differences in mortality.

### Regression analysis

Findings from the multiple regression analysis without any evacuation history data indicated that mortality after the earthquake increased by a factor of three in Facility 1 (Table 5). The interaction term for facility and earthquake suggests significant differences in post-earthquake mortality between facilities. Facility-specific hazard ratios with confidence intervals are shown in Table 6 and indicate that Facilities 1, 2 and 4 experienced significantly elevated mortality after the earthquake.

**Table 5:** Multiple regression model of survival by period.

Variable	Hazard ratio	95% Confidence interval	T statistic	P-value
Facility Number				
1	1.00	NA		
2	0.76	0.50 to 1.14	−1.32	0.2
3	1.01	0.70 to 1.45	0.07	0.9
4	1.20	0.85 to 1.68	1.04	0.3
5	1.04	0.72 to 1.52	0.21	0.8
Sex				
Male	1.00	NA		
Female	0.72	0.55 to 0.96	−2.28	0.02
Age				
50–69	1.00	NA		
70–79	1.37	0.61 to 3.09	0.77	0.4
80–89	1.79	0.84 to 3.80	1.52	0.1
90+	3.11	1.46 to 6.62	2.95	0.003
Care Level				
Low/moderate	1.00	NA		
High	2.05	1.60 to 2.63	5.65	<0.001
Earthquake				
Before	1.00	NA		
After	2.88	1.74 to 4.76	4.13	<0.001
Facility-earthquake interaction				
1	1.00	NA		
2	0.83	0.40 to 1.74	−0.48	0.6
3	0.48	0.22 to 1.05	−1.83	0.07
4	0.82	0.46 to 1.47	−0.66	0.5
5	0.27	0.11 to 0.65	−2.88	0.004

**Table 6:** Post-earthquake facility-specific hazard ratios.

Facility Number	Facility-specific hazard ratio	95% Confidence interval
1	2.88	1.74 to 4.76
2	2.40	1.24 to 4.67
3	1.39	0.69 to 2.81
4	2.37	1.49 to 3.76
5	0.77	0.34 to 1.76

Table 7 shows results of the Cox multiple regression analysis with evacuation history. After adjusting for facility, age, care level, sex and evacuation distance, initial evacuation had twice the mortality of subsequent evacuations. Evacuation distance had no significant impact on mortality, indicating that regardless of length of the evacuations a lot of the residents died after the initial evacuation, and/or that more resilient residents who survived it could also have survived subsequent evacuations.

**Table 7:** Multiple regression model of survival by evacuation characteristics.

Variable	Hazard ratio	95% Confidence interval	T statistics	P-value
Facility Number				
1	1.00	NA		
2	0.59	0.28 to 1.26	−1.37	0.2
3	0.46	0.21 to 1.02	−1.91	0.06
4	0.90	0.27 to 3.30	−0.16	0.9
5	0.12	0.03 to 0.47	−3.08	0.002
Sex				
Male	1.00	NA		
Female	0.70	0.40 to 1.22	−1.25	0.2
Age				
50–69	1.00	NA		
70–79	0.58	0.15 to 2.29	−0.78	0.4
80–89	0.83	0.26 to 2.68	−0.31	0.8
90+	1.81	0.56 to 5.90	0.99	0.3
Care Level				
Low/moderate	1.00	NA		
High	2.09	1.33 to 3.28	3.20	0.001
Evacuation distance (km)				
<150	1.00	NA		
> = 150 & <300	1.01	0.35 to 2.91	0.02	1.0
> = 300	0.92	0.41 to 2.07	−0.19	0.8
Evacuation type				
Initial	1.94	1.07 to 3.49	2.20	0.03
Subsequent	1.00	NA	−0.48	0.6

## Discussion

This study — the first assessment on the health impact of the evacuation after the Fukushima Dai-ichi nuclear accident — showed that under very different disaster conditions, elderly homes in Minamisoma experienced higher increases in mortality risk than US nursing homes that evacuated in the wake of Hurricane Katrina[12], but that increases in mortality were highly dependent on facility-specific factors. Significant increases in mortality after the earthquake were shown in three facilities, and the initial evacuation was associated with twice as many deaths as subsequent evacuations.

There was also a substantial difference in mortality risks across facilities. These differences may be affected by factors such as residents' psychological state or health condition at the time of evacuation, facility-specific evacuation patterns, and the conditions in evacuation sites to which elderly evacuees were admitted[13], [14]. Evacuation distance did not show a significant influence on mortality in the present study. But it was not possible to investigate with certainty whether increases in mortality were due to generalized stress from the earthquake[17]–[19], facility-specific evacuation processes or care quality at evacuation sites because there was no non-evacuated control. According to the interview results with the facility presidents (Table 2), both facilities 4 and 5 evacuated to a distance of about 200 km with support from the government about two weeks after the nuclear accident, but mortality rates were quite different. Facility 4 is an intensive care home for the elderly, whose residents are constantly in and out of the hospital, and this facility's president thought that evacuation might have imposed a higher burden on its residents than in Facility 5, which also had onsite medical services. Facilities 1, 2 and 3 evacuated their residents to areas 300 km or more from Minamisoma city immediately after the nuclear accident without any support from the government. Because of this unplanned relocation, facilities in the evacuation area were not prepared for the evacuees' care: residents had only simple floor mattresses (Japanese *futon*) and medical supplies ceased for three days. This, rather than evacuation distance itself, might explain the high mortality after the initial evacuation; however, it is difficult to measure the quality and continuity of care quantitatively in evacuation sites because no reliable records exist from that period.

Before the earthquake, the Ministry of Internal Affairs and Communications conducted a national survey to investigate prefectural support for disaster management plans for elderly people. This survey assessed whether facilities had an evacuation plan in accordance with the Evacuation Guidelines for Disaster Management[20]. In 2006, 54 of 59 municipalities (91.5%) in Fukushima prefecture reported that they had formulated evacuation strategies[21]. These strategies comprised a five point system in cooperation with the prefectural government: (1) development of an information communication system among disaster-mitigation organizations and social welfare institutions; (2) sharing of elderly residents' data among responsible agencies; (3) implementation of evacuation planning; (4) establishment of support systems in evacuation sites; and (5) coordination and cooperation of relevant organizations in times of disaster[20]. Our findings, however, reveal that the preparation level for a major disaster varied widely between facilities and furthermore, in reality some facilities did not coordinate evacuations with the prefectural government in Minamisoma.

This study had several limitations. There was potential underestimation of relative mortality risk after the earthquake in the Cox proportional hazards analyses, because Facility 4 lacked data on residents who left the facility before the earthquake, which would result in overestimates of incidence density before the earthquake and subsequent underestimation of the relative mortality risk after the earthquake. In addition to this, because there was only one evacuation in Facility 5 it is difficult to compare this facility with the remaining four due to lack of reference to the initial evacuation. However, a sensitivity analysis excluding Facility 5 indicated little influence of this limitation on the regression analysis results. Therefore, the finding that initial evacuation is the most dangerous appears to be robust. Another limitation is that only five of eight nursing homes were involved in this study: one facility lost the residents' records during the tsunami, one had insufficient data for inclusion in the study, and one refused to participate. Because the facilities recorded health information intermittently and/or outsourced health care to external providers, it was not possible to obtain a comprehensive picture of the residents' level of physical health. Unfortunately, the chaotic situation in the prefecture at the time and the rapid reduction in service providers within Minamisoma made obtaining health records from diverse providers within Minamisoma impossible[22]. Thus the confounding effect of poor health on mortality risk during evacuation can only be inferred at a facility level and adjusted for through the fixed effects model, and it is possible that a more refined set of confounders would enable a better understanding of individual-specific risk factors. Future studies on the impact of forced evacuation on the general elderly population are needed to generalize our findings, and to better understand these facility-level influences, such future studies should include detailed interviews and other forms of qualitative research to establish the context in which evacuation mortality occurred.

The necessity of evacuation of vulnerable residents in a post-disaster setting is a controversial issue[9]. The rarity of radiation disasters means that, to date, findings on evacuation-related mortality have been confined to more conventional storm- or earthquake-related disasters. In such settings, such as the aftermath of hurricane Katrina, the decision about whether to evacuate was based on the viability of sheltering in place given the available resources, but in Fukushima the decision to evacuate was at least partly driven by concerns about radiation risk[23] even though there has been no evidence of acute radiation syndrome occurring in residents living in radiation affected areas, or even of high levels of internal exposure[24]. Evacuation has adverse effects, not only on mortality but also clinical status relevant to lifestyle diseases, and leads to an increase in cardiovascular events or other chronic disease sequela[19]. Despite this, fear of radiation exposure in the affected area was severe enough to make evacuation inevitable: almost all residents of Minamisoma city evacuated in a relatively short period. Questions, therefore, about the safety of evacuation of elderly residents and how best to balance the competing risks of radiation exposure and evacuation mortality are of paramount importance. Where the severity of infrastructure collapse and structural damage does not in itself warrant evacuation, careful judgment needs to be exercised in deciding the risk of mortality due to radiation, as it is possible that the evacuation process itself will yield higher mortality than can be expected from radiation exposure. The need for this balancing of risks may apply even in situations where it may ultimately be judged unsafe for residents to return to the affected area, since delays in evacuation, or staggering of evacuation between different institutions on the basis of evacuation mortality risk and preparedness, may lead to significant reductions in mortality due to the initial evacuation process. In this respect, radiation-related evacuations differ from storm-related evacuations, since there may be little or no infrastructure damage in the former, and with proper preparation and support elderly care homes may be able to shelter in place for sufficient time to adequately prepare evacuation sites and mechanisms, and thus reduce the burden of mortality.

In a post-disaster situation where infrastructure collapse affects the essential conditions for maintaining elderly peoples' health[25], [26], evacuation may be essential regardless of the environmental risks posed by radiation exposure. However, the findings of this study indicate that evacuation may not be the best life-saving strategy. In-site relief and care should also be considered as an alternative strategy for disaster planning[27]. Although the Japanese government had issued guidelines for the evacuation strategy and most facilities had been assessed positively, their preparations were not necessarily sufficient to meet the challenges of this triple disaster. The USA maintains a system of regular monitoring and oversight, including fines for breaches and insufficient preparation[13], but the same degree of oversight is lacking in Japan and enforcement mechanisms have not been established: in consequence of this a 2011 review of Japanese facilities found many lacked detailed plans[28]. The national government should consider urgently updating its requirements of nursing homes, reviewing current plans, and strengthening monitoring systems to ensure all areas of the country learn from the lessons of Minamisoma and are prepared for the worst possible contingencies.

This study shows that even under the extreme circumstances experienced in the aftermath of the Great East Japan Earthquake and subsequent radiation accident, some facilities were able to ensure that their residents suffered no significant increase in mortality risk. Balancing the competing risks of radiation exposure and evacuation mortality is of paramount importance when infrastructure collapse and damage do not themselves warrant evacuation. Health planners, disaster coordinators and facility managers in areas that may be subject to similar disasters should consider the lessons of Minamisoma, Fukushima when developing their own plans for disaster response.
